# Identification of Enzymatic Bottlenecks for the Aerobic Production of Malate from Glycerol by the Systematic Gene Overexpression of Anaplerotic Enzymes in *Escherichia coli*

**DOI:** 10.3390/ijms22052266

**Published:** 2021-02-25

**Authors:** Zamira E. Soto-Varela, Gema Cabrera, Agustin Romero, Domingo Cantero, Antonio Valle, Jorge Bolivar

**Affiliations:** 1 Department of Biomedicine, Biotechnology and Public Health-Biochemistry and Molecular Biology, Campus Universitario de Puerto Real, University of Cadiz, 11510 Puerto Real, Spain; zamira.soto@uca.es (Z.E.S.-V.); aguromerovarga@gmail.com (A.R.); 2 Department of Chemical Engineering and Food Technology, Campus Universitario de Puerto Real, University of Cadiz, 11510 Puerto Real, Spain; gema.cabrera@uca.es (G.C.); domingo.cantero@uca.es (D.C.); 3 Faculty of Basic and Biomedical Sciences, Universidad Simón Bolívar, 080020 Barranquilla, Colombia; 4 Institute of Viticulture and Agri-Food Research (IVAGRO)—International Campus of Excellence (ceiA3), University of Cadiz, 11510 Puerto Real, Spain; 5 Institute of Biomolecules (INBIO), University of Cadiz, 11510 Puerto Real, Spain

**Keywords:** C4 compounds, malate, succinate, glycerol, TCA cycle, anaplerotic enzymes, Pck, bicarbonate, *Escherichia coli*

## Abstract

The biotechnological production of dicarboxylic acids (C4) from renewable carbon sources represents an attractive approach for the provision of these valuable compounds by green chemistry means. Glycerol has become a waste product of the biodiesel industry that serves as a highly reduced carbon source for some microorganisms. *Escherichia coli* is capable of consuming glycerol to produce succinate under anaerobic fermentation, but with the deletion of some tricarboxylic acid (TCA) cycle genes, it is also able to produce succinate and malate in aerobiosis. In this study, we investigate possible rate-limiting enzymes by overexpressing the C-feeding anaplerotic enzymes Ppc, MaeA, MaeB, and Pck in a mutant that lacks the succinate dehydrogenase (Sdh) enzyme. The overexpression of the TCA enzyme Mdh and the activation of the glyoxylate shunt was also examined. Using this unbiased approach, we found that phosphoenol pyruvate carboxylase (Ppc) overexpression enhances an oxidative pathway that leads to increasing succinate, while phosphoenol pyruvate carboxykinase (Pck) favors a more efficient reductive branch that produces mainly malate, at 57.5% of the theoretical maximum molar yield. The optimization of the culture medium revealed the importance of bicarbonate and pH in the production of malate. An additional mutation of the *ppc* gene highlights its central role in growth and C4 production.

## 1. Introduction

The Department of Energy of the United States and the European Commission have included the four-carbon (C4) dicarboxylic acids, succinate, malate, and fumarate, among the top 12 selected building blocks [1,2]. For instance, malic acid is used in the food industry and in the synthesis of several organic fine chemicals [3,4], such as pharmaceutical compounds and new polymers [5]. However, their traditional production, based on petrochemicals, harms the environment, and therefore they should be produced by biotechnological processes.

*Escherichia coli* is one of the microorganisms most widely used as biocatalyst in metabolic engineering. Under anaerobic conditions, *E. coli* produces a mixture of succinate, formate, acetate, lactate, and ethanol to maintain redox balance; however, the wild-type strains are not capable of producing malate or fumarate. Succinate is the end product of the naturally occurring reductive pathway, in which phosphoenolpyruvate (PEP) accumulation is a fundamental issue for C4 production. Two enzymes are potentially capable of driving PEP carboxylation to produce oxaloacetate (OAA); on the one hand, PEP carboxylase (Ppc) is the main component responsible for this reaction in *E. coli* during glucose fermentation. This is, in basic terms, an irreversible reaction that wastes the high-energy phosphate of PEP [6]. In contrast, the PEP carboxykinase (Pck) catalyzes the reversible carboxylation of PEP to OAA, preserving energy as ATP. However, *pck* gene expression is repressed at high glucose concentration in *Escherichia coli* and is only activated to operate in a reverse direction for gluconeogenesis during the oxidative metabolism of organic acids [7,8]. Additionally, the malic enzymes MaeA and MaeB (NADH- and NAPDH-dependent, respectively) are also reported as gluconeogenic and repressed by glucose, catalyzing the reversible carboxylation of pyruvate to malate [7,9]. On the other hand, the oxidative branch, which diverts OAA and Acetyl-CoA to isocitrate, operates at a low rate to produce α-ketoglutarate for NH_4_ assimilation [10].

Several metabolic engineering strategies have been developed to redirect metabolic fluxes toward C4 biosynthesis both in anaerobiosis and in aerobiosis. These strategies usually involve the inactivation of competitive pathways such as ethanol, lactate and acetate production. To further rewire C-flux, activation of the glyoxylate shunt and overexpression of anaplerotic enzymes have been also carried out [11,12]. For instance, Zhang et al. [13] reported that a high expression of the *pck* gene improves the fermentative production of succinate significantly. A similar strategy has been applied for succinate production from glycerol in a *ldhApflB* mutant [14]. Zhang et al. [13] also reported that an additional deletion of the fumarate reductase genes (*frdBC*) alone was sufficient to redirect carbon flow into malate under anaerobic conditions. Additional deletions of fumarase (*fumA, B, C*) and malic enzyme genes increased malate yield up to 1.42 mol malate/mol glucose (34 g/L) in a two-stage process (aerobic cell growth and anaerobic malate production) [15]. Co-overexpression of malate dehydrogenase (Mdh) and Pck enzymes is also reported to promote anaerobic production of malate in a similar mutant background [16]. Moon et al. [17] combined the use of *Manheimia succiniciproducens* phosphoenolpyruvate (PEP) kinase (PCK), which converts PEP into oxaloacetate (OAA) more efficiently than the autologous enzyme, with the blockage of acetate (*△pta*) obtaining 0.75 mol malate/mol glucose (9.25 g/L) of malate. On the other hand, recent work [18] has linearized the TCA cycle by deleting the *mdh* and *mqo* gene and activated the glyoxylate shunt by deleting the *iclR* gene. They also deleted the *maeA* and *maeB* genes and expressed a malate-insensitive Ppc and a NADH-insensitive citrate synthase (GltA) to obtain a 0.82 mol malate/mol glucose yield from glucose. In another approach, Gao et al. [19] used in vitro modular pathway optimization combined with in vivo multiplexed combinatorial engineering using CRISPR interference to identify a malate biosynthetic pathway. They engineered the strain *△adhE△ackA-pta△ldhA△maeA△maeB△mdh△iclR△arcA*, which, together with controlled expression of the pyruvate carboxylase (Pyc) and citrate synthase (GltA) enzymes, yielded 0.85 mol malate/mol glucose in shake-flask culture [19].

Most of the previous works describing malic acid production in *E. coli* use glucose as the carbon source in a process that competes with food production. It is therefore essential to use renewable alternative carbon sources, such as xylose [20]. Glycerol is becoming an inexpensive feedstock because of the global increase in bio-diesel production (www.ebb-eu.org accessed on 10 January 2021) [21]. Glycerol is a higher-reduced carbon source compared with glucose, and therefore each glycerol molecule could be converted to reduced compounds such as C4 and maintain redox balance. However, no studies have been published on malate production using glycerol as the carbon source in *E. coli* in aerobic conditions. In the present work, we explore this approach through two strategies: (1) a study on putative bottlenecks by measuring the extracellular organic acids from two genetic background strains overproducing the autologous anaplerotic Ppc, Pck, MaeA and MaeB enzymes and the malate dehydrogenase Mdh, which can potentially produce succinate, malate and fumarate in aerobiosis and (2) the design of an experiment (D.O.E) to study the influence of culture medium components in the production of malic acid in the most appropriate engineered *E. coli* strain.

## 2. Results

### 2.1. Overexpression Screening of Malate Dehydrogenase and Anaplerotic Enzymes in E.coli M4 and M4-ΔiclR Mutant Strains

**Figure 1:**
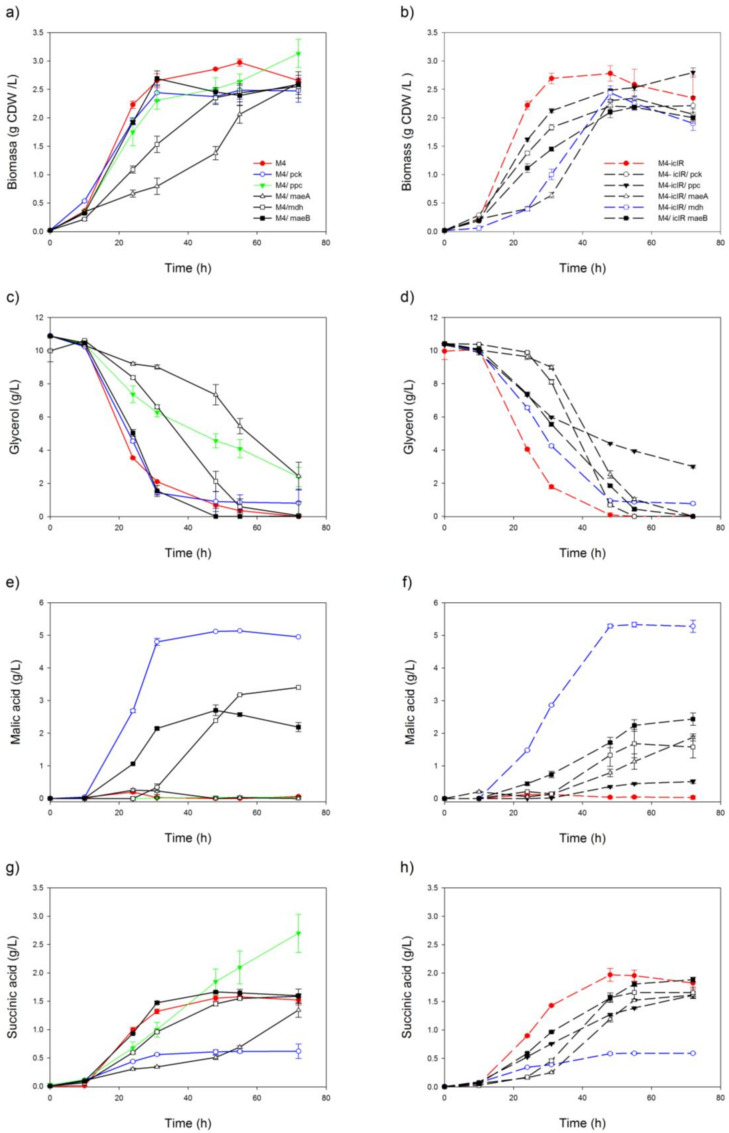
Curve plots of parameters measured in a screening of anaplerotic and cataplerotic overexpression on the engineered M4 (**a**,**c**,**e**,**g**) and M4-Δ*iclR* strains (**b**,**d**,**f**,**h**). Biomass growth in cell dry weight (CDW)/L (**a**,**b**). Glycerol not consumed in g/L (**c**,**d**). Malic acid production in g/L (**e**,**f**) and succinic acid production in g/L (**g**,**h**). All these parameters were evaluated at 0, 10, 24, 31, 48, 55 and 72 h. The overexpression’s enzymes cloned in pBAD vectors were induced with 0.02% L-arabinose.

**Figure 2:**
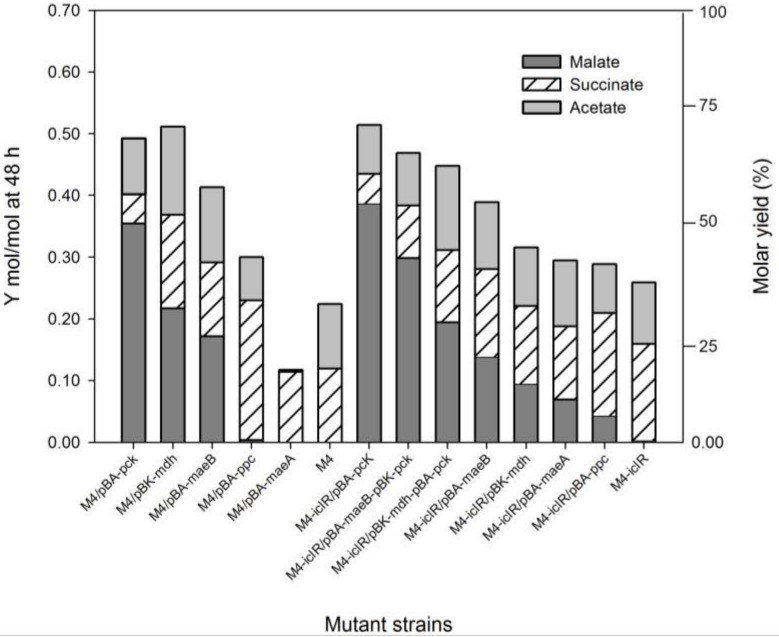
Cumultive bar charts of C4 metabolites and acetate molar yields in mol/mol (*Y* right axis) and molar yields in % (*Y* left axis) of overexpression and co-expression on M4 and M4-Δ*iclR* strains at 48 h. Standard deviation for all parameters was <0.04.

Two mutant strains were constructed for the aerobic production of C4. In one of them (M4 strain), the synthesis of acetate was confined by deletion of the acetate kinase–phosphotransacetilase (*△ackA-pta*) and pyruvate oxidase (*△pox*) genes. The TCA cycle was also split into two lineal pathways by deletion of the succinate dehydrogenase (*sdhA*) gene, which codifies for one of two catalytic subunits (the FAD-binding protein), impairing the synthesis of fumarate from succinate. In this mutant background, succinate is the end product of the oxidative pathway, while malate and fumarate could be generated in the reductive branch. To study the effect of the activation of the glyoxylate shunt on C4 production, the repressor gene *iclR* was also deleted in a second strain (M4-Δ*iclR* strain). In order to discover putative bottlenecks, the C4 and acetate were measured in the supernatant samples from different strains overproducing the autologous anaplerotic enzymes Ppc, Pck, MaeA and MaeB and the TCA enzyme malate dehydrogenase Mdh. All these strains were grown in a defined culture medium containing 10.5 g/L glycerol as a carbon source, and the extracellular concentrations of malate, succinate, glycerol, acetate and biomass were evaluated for up to 72 h (Figure 1). Malate, succinate and acetate molar production yields (mol/mol glycerol consumed) (Figure 2) and g/g (Figure S1) were plotted after 48 h. Fumarate was not detected by High Performance Liquid Chromatography (HPLC) in any of the assayed conditions. Analysis of the M4 and M4-Δ*iclR* mutant strains shows that both strains were capable of producing succinate, although the activation of glyoxylate shunt in M4-Δ*iclR* slightly increased the production (from 1.5 to 1.7 g/L, respectively) and yield (Figure 1g,h) from 0.11 to 0.17 mol/mol yield (Figure 2), though no malate was detected. Regardless of *ackA-pta* and *pox* gene deletions, a small amount of acetate was found in all of the conditions assayed (0.09–0.155 mol/mol), except M4/pBA-*maeA*, in which no acetate was detected (Figure 2). In our screening, the overexpression of Pck, Mdh, MaeA, MaeB and Ppc enzymes using the inducible pBAD vector had an effect on malate and/or succinate production in most cases, however, malate was the C4 produced at the highest concentration (Figure 1f) and yield (Figure 2) compared with succinate (Figure 1h and Figure 2). It is worth noting that although Pck and Ppc catalyzed the same conversion (PEP→OAA), they had a remarkably different effect on C4 production. Thus, Ppc overexpression in M4 improved 80% of succinate production (Figure 1g), but no malate was detected (Figure 1e). However, Pck had the opposite effect in both mutants because most of the consumed glycerol (Figure 1c,d) was transformed into malate (5.1–5.3 g/L at 55 h) (Figure 1e,f), while succinate production was less than half that of the M4 and M4-Δ*iclR* strains (Figure 1g,h). Mdh overexpression also increased malate production in both strains, although to a lesser extent than Pck and maintaining the same proportion of succinate (Figure 1e,f and Figure 2). The overexpression of malic enzymes A and B also showed different behavior because overexpression of MaeB increased malate production and yields, but MaeA had not a remarkable impact on C4 production in either the M4 strain or M4-Δ*iclR* (Figure 1e,f and Figure 2).

Nevertheless, the co-overexpression of Pck and Mdh or MaeB in M4-Δ*iclR* has no synergic effect on the synthesis of malate because malate yield decreases with respect to the single overexpression of either of them (Figure 2). The glyoxylate shunt, on the other hand, does have a synergic effect with Pck overexpression on malate production because the conversion rate for malate is 10% higher in M4-Δ*iclR*/pBA-*pck* in respect to that obtained for the M4/pBA-*pck* strain (Figure 1e,f and Figure 2), although succinate was exported at the same rate (Figure 1g,h and Figure 2). It is worth noting that in this strain, glycerol was not completely exhausted, with the remaining 10% of glycerol being unconsumed (Figure 1c,d). Overexpression of the different enzymes assayed in both mutant strains also significantly affected cell growth. Thus, the highest growths were observed in mutants without overexpression, although M4-Δ*iclR*/pBA-*pck* showed similar growth trends (Figure 1a,b). Due to its high concentration and yield for malate production, M4-Δ*iclR*/pBA-*pck* was selected for optimization of the culture medium.

### 2.2. Identification of the Culture Media Factors That Affect to the Malate Production

To study the culture media factors that significantly affect to malic acid production in the M4-Δ*iclR/*pBA-*pck* strain, the effect of the concentration of 10 components of the culture medium, together with L-arabinose, the inducer of Pck overexpression by the pBAD vector, were evaluated using the Plackett-Burman design (Table 1).

**Figure 3:**
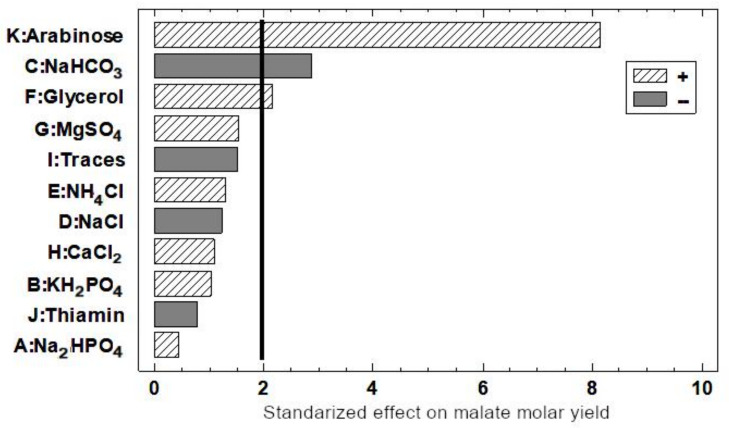
Pareto bar chart of medium culture components screening using malate yield (mol malate per mol glycerol consumed) at 48 h as a response variable. The bars of components that have a negative effect on malate yield are grey and those of components that have a positive effect on malate yield are filled in with diagonal lines. The vertical black line represents the statistically significant value with a 95% confidence interval.

To this aim, a diagram of malic acid yield (mol/mol) with respect to the glycerol assimilated (Figure 3) was performed as described in the experimental procedure section. As can be observed in Figure 3, L-arabinose was the factor that most positively affected malate yield, with a confidence level of 95%. Nevertheless, other factors like the bicarbonate (negatively) and glycerol (positively) were also relevant to improving malate yield.

### 2.3. Optimization of Culture Conditions

#### 2.3.1. Effect of Pck Overexpression Level on Malate Production

Considering that the L-arabinose is the factor that mostly affected malate yield, a sweep of different L-arabinose concentrations was assayed, and the parameters molar yield, productivity, specific production and specific productivity of malate were calculated (Table S2). According to these results, a range between 0.025–0.05% showed the best values for most parameters.

#### 2.3.2. Kinetic Study of Glycerol Consumption and Malate Production

As mentioned above, another factor that significantly affected malate production was glycerol concentration in the culture medium. For the optimization of this parameter, several concentrations of glycerol (from 3.5 up to 37.5 g/L) in the culture medium were evaluated to study the kinetic of carbon source consumption (Figure S2). The cell growth was proportional to the initial glycerol concentration (Figure S2A), and the highest growth occurred with 31 and 37 g/L of glycerol (2.35 CDW/L at 48 h). However, at lower concentrations (3.5 or 6.5 g/L of glycerol) specifically, the carbon source was completely consumed (Figure S2B). Likewise, the malic acid produced was also related to the initial glycerol concentration, and 12.5 g/L of glycerol was the optimal initial concentration (Figure S2C).

#### 2.3.3. Effect of Sodium Hydrogen Carbonate on Malate Production

**Figure 4:**
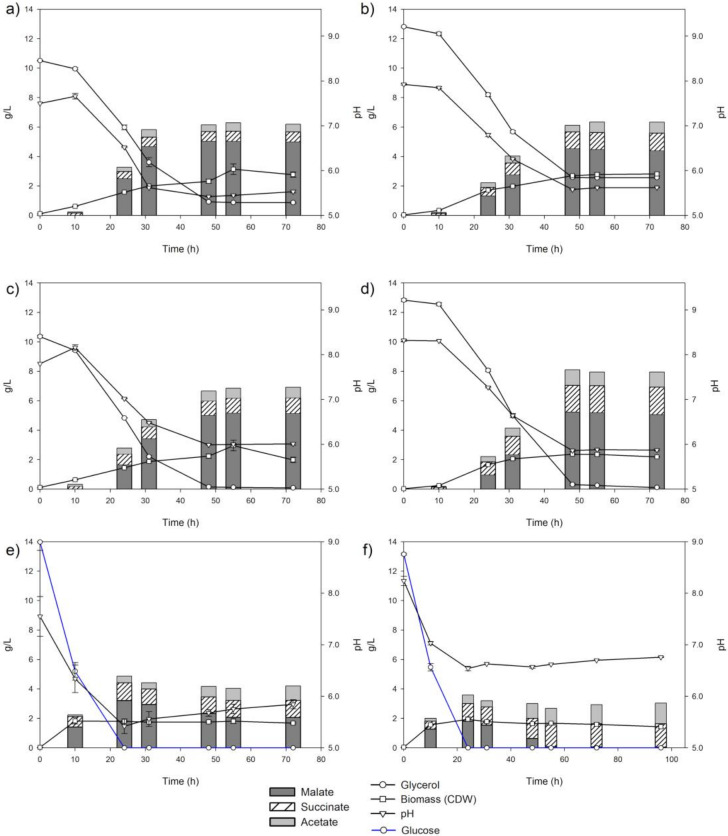
Bicarbonate and glycerol assays with several initial concentrations: 2 g/L of bicarbonate and 10 g/L of glycerol (**a**); 2 g/L of bicarbonate and 12.5 g/L of glycerol (**b**); 4 g/L of bicarbonate and 10 g/L of glycerol (**c**); 4 g/L of bicarbonate and 12.5 g/L of glycerol (**d**); 13 g/L of glucose with 2 g/L of bicarbonate (**e**); and 13 g/L of glucose with 4 g/L of bicarbonate (**f**). Standard deviation of succinic acid, malic acid and acetic acid are shown in Table S3.

Pareto Diagram analysis indicated that bicarbonate was the second factor that more significantly affected the malate yield (Figure 3). Bicarbonate concentration in the culture medium was established at 2 g/L for previous experiments in the screening (10.5 g/L of glycerol, Figure 2) and for glycerol optimization (12.5 g/L of glycerol). To study the influence of bicarbonate, we tested these glycerol concentrations using 4 g/L of bicarbonate, and we evaluated malate, succinate and acetate as well as pH, glycerol and CDW/L up to 72 h (Figure 4). We found that this increment of bicarbonate improved glycerol assimilation, which was almost completely consumed at 48 h for both glycerol concentrations. This additional glycerol was transformed into C4 compounds (malate and succinate) and exported because cell growth did not change, and the C4 compounds increased at both glycerol concentrations (Figure 4c,d). Thus, the fermentation with 4 g/L of bicarbonate and 12.5 g/L of glycerol led to the highest concentrations of the accumulated C4 compounds—malate (5.25 g/L) and succinate (1.79 g/L)—at 48 h (Figure 4d). Considering acetate exported acetate (1.05 g/L), conversion from consumed glycerol to exported carbon compounds (C4 plus acetate) represents a 65% yield (g/g). During fermentation with 10.5 g/L of glycerol, malate and acetate production was similar, but succinate concentration in the culture medium was lower (Figure 4c). Therefore, the increment of bicarbonate seems to favor the redirection of the carbon source to succinate instead of malate (Figure 4c,d). This bicarbonate concentration (4 g/L) was the optimal for glycerol assimilation and C4 production because the absence of bicarbonate (Figure S3A) or the addition of 2 g/L to the culture medium led to lower values of C4 production (Figure 4a,b). Alternatively, with the addition of 6 g/L of bicarbonate, the M4-Δ*iclR*/pB-*pck* strain was unable to grow and produce C4 compounds, probably because at this latest concentration pH was too high (Figure S3B). Thus, the initial pH increased with bicarbonate concentration (7.25, 7.92, 8.32 and 8.60 for 0, 2, 4 and 6 g/L of bicarbonate, respectively) (Figure 4 and Figure S3). In all conditions, but not for 6 g/L of bicarbonate, pH values dropped near to 6 (5.5, 5.7 and 5.9 for 0, 2 and 4 g/L of bicarbonate, respectively). In order to test the relevance of the optimization of both strain and culture conditions for the use of glycerol as a carbon source, glycerol was replaced by 13 g/L of glucose in the M9 medium, supplemented with 2 and 4 of g/L of bicarbonate, and assayed with the M4-Δ*iclR*/pB-*pck* strain (Figure 4e,f). Although glucose was rapidly consumed at 4 g/L of bicarbonate, only 2 g/L of malic acid were produced at 24 h. What is more, contrary to what happens when glycerol is the carbon source, the malic acid produced at 24 h was consumed completely at 55 h (Figure 4f). However, at 2 g/L, the exported malate is not completely consumed (Figure 4e). Interestingly, this behavior correlates with a different pH in the culture medium, which only dropped to 6.7 with 4 g/L of bicarbonate, instead of 5.7 in the case of glycerol or 5.5 in the case of glucose with 2 g/L of bicarbonate (Figure 4e). Due to the importance of bicarbonate in glycerol consumption and malate production, several experiments were carried out adding bicarbonate along the biotransformation process in different feeding configurations with 12.5 g/L of glycerol, as shown in Figure S4. As can be observed, in all of these conditions, glycerol was almost completely consumed, but none of them improved malic acid production, although succinate was increased in all of the conditions (Figure S4). It is worth noting that in all cases with glycerol, the pH stabilized above 6 from 48 h onwards (Figure 4). We reasoned that a key element could be not only bicarbonate itself but also the pH of the culture medium. The addition of 4 g/L of bicarbonate helps to maintain the pH at around 5.9, which would be the optimal pH condition. To address this question, biotransformation assays were carried out using M9 medium supplemented with 2 g/L of bicarbonate and but keeping pH at around 5.9 by adding NaOH (Figure S5). As can be observed, glycerol consumption and malic and succinic acids production increased with respect to those of the biotransformation using 2 g/L of bicarbonate. Thus, glycerol was completely consumed at 55 h, and malate and succinate concentrations reached 4.75 g/L and 1.48 g/L, respectively, at 72 h, which is an intermediate output between the addition of 2 and 4 of g/L of bicarbonate, although in this case, acetate concentration was slightly higher.

**Figure 5:**
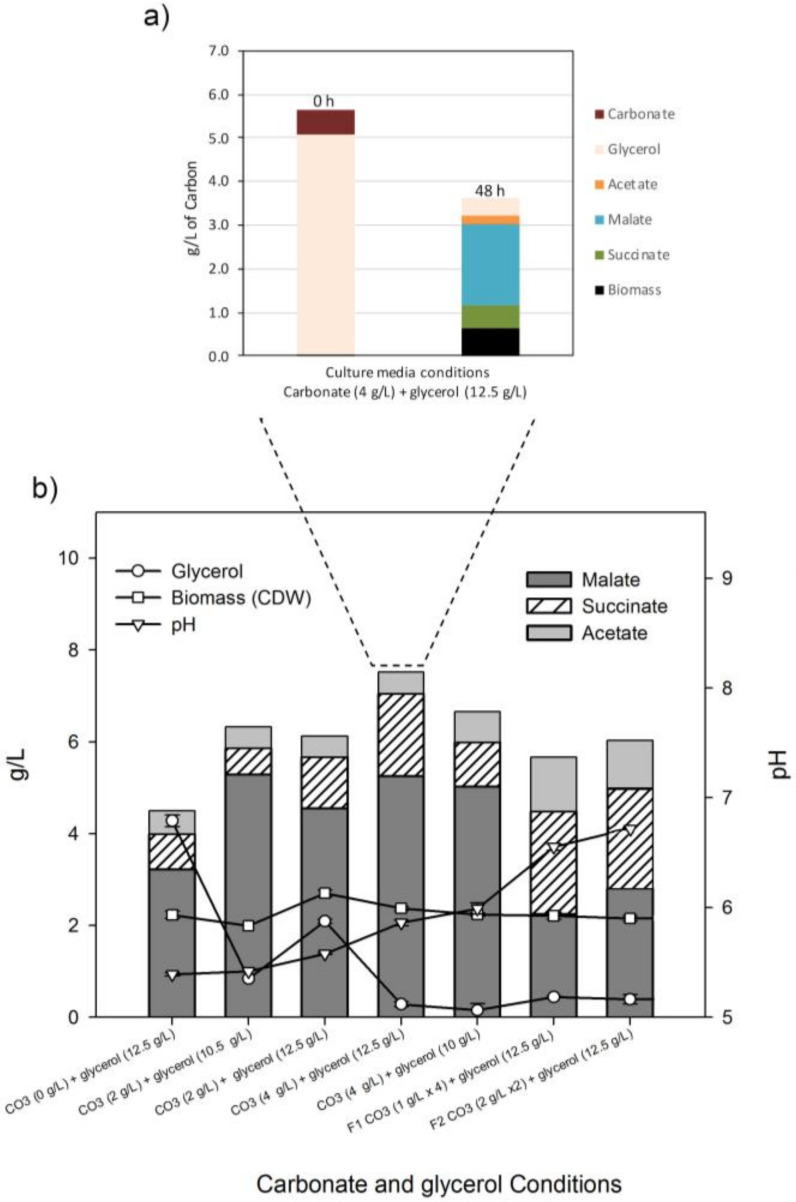
Elemental C analysis (**a**) and summary of bicarbonate and glycerol concentration assays at 48 h (**b**).

The importance of pH in the ratio of malate/succinate production is highlighted in Figure 5b, where the concentration of both compounds at 48 h in all the conditions assayed in this work are represented together with pH. As can be observed, the proportion of malic acid was higher when pH was lower than 6, and the concentration of both compounds tended to be similar when pH was above this value. Using this condition, elemental C analysis allowed us to determine bicarbonate assimilation and total organic acids production at 48 h (Figure 5a). The C content in the initial M9 medium was 5.6 g/L (glycerol and bicarbonate), while the C measured after 48 h was 0.66 g/L in the biomass and 2.97 g/L in the supernatant of the culture medium, which represented the sum of the C content of the compounds detected by HPLC (Table S4). Therefore, the initial bicarbonate in the culture medium (4 g/L) was consumed to produce succinate and malate. As can be expected, there is a significant amount of C released as CO_2_ (~2 g/L) through decarboxylation reactions in the TCA oxidative branch, the amino acids catabolism, and the pentose phosphate pathway.

#### 2.3.4. Ppc Deletion on the M4-Δ*iclR* Genetic Background Hinders Cell Growth

We reasoned that given that Ppc improves the oxidative pathway and Pck increased malate production through the reductive branch, the deletion of the *ppc* gene (M6 strain) could more efficiently rewire PEP into malate, lowering succinate co-production. However, this mutant was not able to grow on glycerol (Figure S6) or glucose (data not shown) minimal medium. Growth was rescued by overexpressing *ppc* (M6/pBA-*ppc*), which was even higher than that of the M4-Δ*iclR* reference strain. Pck, therefore, is not capable of replacing the role of Ppc in aerobiosis, in contrast with the anaerobic fermentative metabolism as reported previously [13].

## 3. Discussion

**Figure 6:**
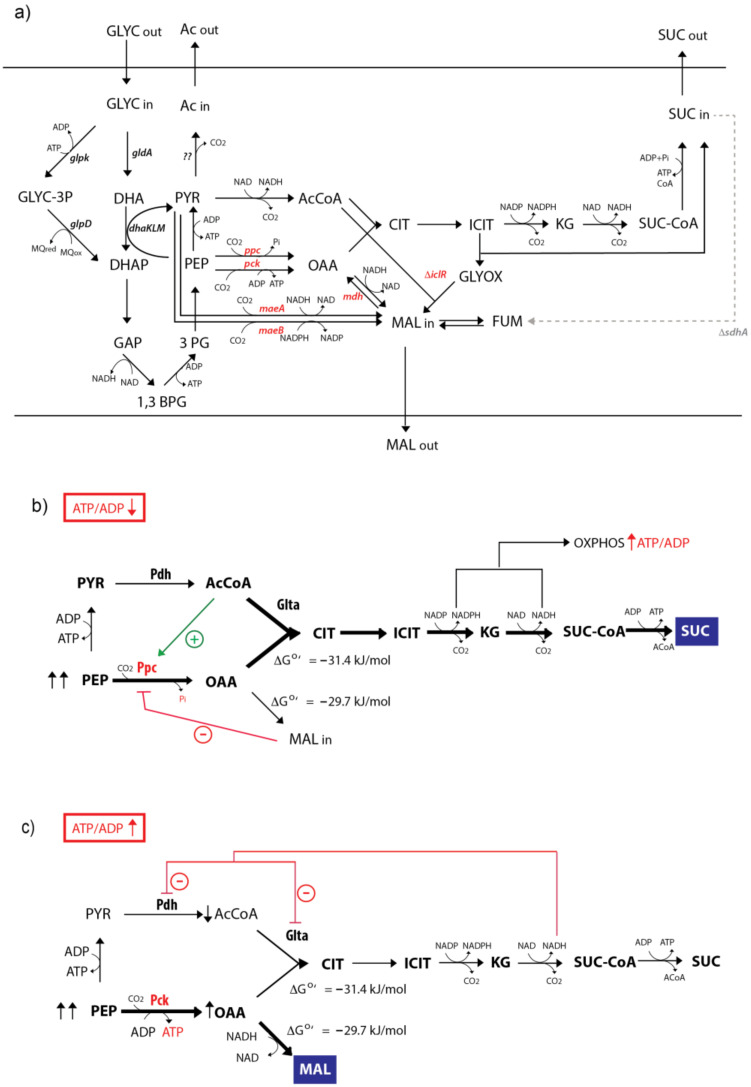
Metabolic diagram of glycerol assimilation and succinate and malate biosynthesis under a TCA cycle split into two branches due to the deletion of the succinate dehydrogenase (*sdhA*) gene (grey dashed line). Arrows in the PYR-PEP-OAA node indicate the net flux for each reaction in the M4-Δ*iclR* strain when the enzyme marked in red is overexpressed. (**a**) Metabolic diagram with the genes overexpressed in this work depicted in red. The *iclR* gene is also highlighted in red to indicate activation of the glyoxylate shunt. (**b**) Metabolic rewiring proposed for overexpression of Ppc; (**c**) Metabolic rewiring proposed for overexpression of Pck. The acronyms are as follows: 3PG: 3-phosphoglycerate, Ac: acetate, AcCoA: Acetyl-CoA, CIT: citrate, DHA: dihydroxyacetone, DHAP: dihydroxyacetone phosphate, FUM: fumarate, Glta: citrate synthase, Glyc: glycerol, GLYOX: glyoxylate, ICIT: isocitrate, KG: 2-α-ketoglutarate, MAL: malate, OAA: oxaloacetate, OXPHOS: oxidative phosphorylation, PEP: phosphoenolpyruvate, Pdh: pyruvate dehydrogenase, PYR: pyruvate, SUC: succinate, SUC-CoA: succinyl-CoA.

Future research directions may also be highlighted. This work aimed to investigate bottlenecks in the aerobic production of C4 from glycerol in an unbiased way and determine the most favorable C4 product. Glycerol is a highly reduced carbon source that can be considered a waste product of the biodiesel industry. Since C4 production requires both ATP and a high redox balance, glycerol can be an optimal precursor. Several studies describe the anaerobic fermentation of glycerol to malate, although the aerobic production of this compound using glycerol has not been described so far. C4 aerobic production in *Escherichia coli* would benefit over anaerobic fermentation in terms of faster biomass generation. Besides, aerobic metabolism has the advantage of favorable phosphate-donor usage for glycerol assimilation (Figure 6a). In the aerobic pathway, ATP is the GlpK cofactor for glycerol phosphorylation, while in the anaerobic metabolism, DhaK uses PEP as a phosphate donor for DHAP synthesis, lowering the PEP pool available for C4 production [22]. As Zhang et al. reported, aerobic assimilation seems to be most appropriate for C4 production form glycerol, even in anaerobiosis, because the deletion of any single gene concerned with the GldA-DhaKLM pathway almost doubles the succinate yield in anaerobic fermentation in a *pck*-unrepressed strain [23]. These authors concluded that the native anaerobic pathway does not appear desirable for succinate production and limits the usage of PEP for OAA production.

The PEP/pyruvate/OAA/malate node is a complex and highly regulated metabolic system. In our approach, we used a mutant (M4) with impaired acetate production and a TCA cycle split into two linear pathways by *sdhA* deletion: an oxidative one with succinate as the end product and a reductive branch that could produce malate and fumarate. By measuring the extracellular organic acids from different enzymes overproducing strains, we were able to analyze the rewire of C4 flux. We reasoned that overexpressing active genes in the aerobic metabolism such as *ppc*, *mdh* and genes that are normally repressed (*pck*, *maeA* and *maeB*) could hint at new strategies for C4 production. *iclR* deletion in this parental strain allowed us to analyze the effect of the glyoxylate shunt activation, with or without the same overproducing genes (Figure 6a).

We found that the M4 mutant produces succinate at a relatively low rate because the oxidative pathway requires two glycerol molecules to generate one succinate. Activation of the glyoxylate shunt (M4-Δ*iclR*) improves succinate production (3 Gly → 2 C4 + CO_2_). Both M4 and M4-Δ*iclR* strains did not accumulate malate, indicating that the malate originating in the glyoxylate shunt was oxidized to OAA, which again entered the oxidative arm of the TCA cycle (Figure 6a and Figure S7A). However, overexpression of any of the anaplerotic enzymes or Mdh leads to an increase in C4. Therefore, a PEP pool is available to generate C4 from a direct carboxylation or via pyruvate. The malic enzyme NADPH-linked MaeB increases malate without affecting cell growth. This fact would denote a high NADPH/NADP^+^ relation that can be used for pyruvate carboxylation (Figure S7B). Additional modifications to activate the oxidative Pentose Phosphate Pathway could increase this ratio to improve malate production (1 Gly → 1 Pyruvate + CO_2_ → 1 Malate). In contrast, NADH-linked MaeA overexpression does not produce any malate and decreases cell growth, probably because the NADH/NAD^+^ ratio drops. Pyruvate carboxylation competed in this case with oxidative phosphorylation and acetyl-CoA synthesis since no acetate was detected (Figure S7C).

Both Ppc and Pck can drive PEP carboxylation into OAA. However, overexpression of these enzymes in the M4 strain led to entirely different outputs. While Ppc overexpression increased the oxidative pathway to succinate, Pck overexpression tilted the balance toward the reductive pathway because most of the glycerol was transformed into malate and exported to the culture medium. The production of C4 is also much more efficient than that of succinate (1 Gly → 1 PEP + CO_2_ → OAA → 1 malate). The regulation of this complex node may explain this different output (Figure 6b,c). OAA, the product of both enzymes, can be reduced to malate by Mdh because the ΔG’° of the reaction OAA to malate is −29.7 kJ/mol; that is, it is an irreversible reaction [24]. Alternatively, OAA can react with acetyl-CoA from by pyruvate decarboxylation to generate citrate (ΔG’° = −31.4 kJ/mol) that leads to succinate through the oxidative pathway. Both reactions are, therefore, thermodynamically favored and irreversible (Figure 6b,c). However, Ppc is a highly regulated allosteric enzyme [25] that is reported to require acetyl-CoA for activation [26] and to be inhibited by malate. For instance, [18] used a malate-insensitive version of this enzyme to implement an oxidative pathway toward malate.

When Ppc is the only enzyme for PEP carboxylation, OAA synthesis from PEP is balanced with the oxidation of PEP to pyruvate and then to acetyl-CoA. The balanced concentrations of malate, acetyl-CoA and more favorable ΔG’° would lead to the synthesis of citrate instead of malate, promoting, therefore, the oxidative pathway (Figure 6b). In contrast, Pck is a monomeric enzyme with no allosteric modulation whose transcription is activated only for organic acids assimilation [7,8]. Therefore, the lack of feedback inhibition in Pck overexpression causes malate accumulation that must then be exported (Figure 6c). On the other hand, Pck increasing activity conserves the high energy of PEP, leading to net production of ATP [1 glycerol → 1 Malate + 2 ATP + PQH_2_ (~2 ATP trough oxidative phosphorylation)]. Kwon et al. [27] reported that intracellular ATP was two times higher in *E. coli* cells overexpressing Pck than in those with or without Ppc overexpression. A high ATP/ADP ratio would inhibit oxidative phosphorylation, and the NADH produced through the oxidative pathway could accumulate, inhibiting GltA and Pdh, which in turn would downregulate the oxidative pathway (Figure 6c). Zhang et al. [13] reported that a high expression of Pck might substitute Ppc’s function in the anaerobic metabolism, enhancing the reductive pathway at the same time. However, this was not possible during aerobic metabolism because the M6 strain was not able to grow on glycerol or glucose, even with Pck overexpression. A possible alternative to increase aerobic malate production would be the replacement of the wild-type *ppc* gene with a less active form modulated through an inducible vector.

It is worth noting that, despite the lack of ack, PTA, and pox genes, acetate was present in the supernatant of all the strains assayed, except in the M4/pBA-*maeA* strain. Therefore, other acetate producing enzymes such as citrate lyase (citrate → acetate + OAA) or aldehyde dehydrogenase (acetaldehyde + H_2_O+ NADP^+^ → acetate + NADPH + 2 H^+^) must be operating. Although the conversion from glycerol to (mainly) malate is considerable in the M4-Δ*iclR*/pBA-*pck* strain, glycerol was not completely consumed. For this reason, we studied the effect of all the components of the culture medium on malate in this strain. In this study, besides glycerol and the inducer of Pck expression, bicarbonate concentration was found the most influential factor. This may be because low affinity for bicarbonate of Pck affects to the catalytic efficiency of PEP carboxylation [27]. Bicarbonate concentration was also an important factor for glycerol assimilation. We found that the optimal concentration was 4 g/L, at which all the glycerol was consumed at 48 h (Figure 4c,d). The C analysis revealed that all bicarbonate in the initial culture medium was consumed. In fact, since the synthesis of 50.38 mM of C4 (malate + succinate) requires the same amount of bicarbonate, the initial 47.62 mM of bicarbonate (4 g/L) was not enough, and extra carbon was incorporated as CO_2_ (Figure 5a). Thus, bicarbonate has two roles, as a pH buffer and as a carbon source, although this strain was able to fix CO_2_ since it was able to produce malate and succinate using an M9 medium without bicarbonate (Figure S3A). On the other hand, when the cells were grown without or with 2 g/L of bicarbonate, pH dropped to 5.5 and 5.6, respectively, at 48 h, remaining stable up to the final time point studied, and glycerol was not completely consumed (Figure 5). However, in a medium containing 2 g/L of bicarbonate but keeping pH at around 6 by adding NaOH (Figure S4), glycerol was also consumed, although at 56 h instead of 48 h. Nevertheless, a pH above 6 negatively affects malate production (Figure 5). This may be due to malate exporters. During aerobic metabolism, DctA and DauA are the reported C4 transporters [28], although it has been recently described that the anaerobic DcuA transporter can also be responsible for malate export [18]. One of the factors that affects C4 export is pH, and for instance, it has been described that pH 6 is optimal for succinate [29]. However, our results suggest that for an optimal malate export, pH must be under 6. Thus, pH seems to be critical for both glycerol assimilation and malate export. Interestingly, when glycerol is replaced by glucose, the M4-Δ*iclR*/pBA-*pck* strain only produces malate up to 24 h when glucose is consumed, and the cells turn into a gluconeogenic mode. However, when the cells grew with 4 g/L of bicarbonate, they were able to import and consume the produced malate (pH 7), while when this strain was cultured at 2 g/L, pH drop to 5.5, and most of the malate remained in the culture medium regardless the gluconeogenic situation. This would indicate that a pH below 5.5 is the most favorable to export malate.

## 4. Materials and Methods

### 4.1. Bacterial Strains and Construction of Knockout Strains

The background mutant used as a host strain for the overexpression screening are the quadruple mutant (M4), the quintuple mutant (M4-Δ*iclR*), and the sextuple mutant (M6) strains, which were constructed from the *sdhA*:kan parental strain, purchased from Keio collection (NAIST, Kyoto, Japan) [30] following the homologous recombination method described by Datsenko and Wanner [31]. All of the gene knockout strains were verified by PCR and the primer pairs, plasmids, and strains used in this study are listed in Tables S5–S7, respectively.

### 4.2. Gene Cloning of Anaplerotic and Cataplerotic E. coli Enzymes and Overexpression Assays through M4 and M4-ΔiclR Mutants

Cloning of the *E. coli maeA, maeB* and *mdh* ORFs in pBAD vectors (pBAD-HisA or pBAD-18-Kan), which allow for inducible expression of the P_BAD_ promoter upon addition L-arabinose to the culture medium [32], were performed using the standard procedures [33] of Velocity^TM^ DNA polymerase (Bioline Reagents Ltd., London, UK) with the primers and plasmids listed in Tables S5 and S6, respectively. The *pck* and *ppc* ORFs cloned in the pBAD/HisA vector were performed in Valle et al. [34]. The resulted clones were checked by PCR, and the selected ones were purified and sequenced with 3730XL DNA sequencer (StabVida, Lisboa, Portugal) and compared with the gene sequences using ClustalW software. DNA isolation (PCR products and plasmids) was performed using purification kits from Macherey-Nagel (GmbH & Co., Düren, Germany).

### 4.3. Growth Conditions

Assays were carried out in 250 mL Erlenmeyer flasks at 37° C in a rotary shaker at 200 rpm using the M9 medium containing in 1 L: 9.97 g Na_2_HPO_4_, 3 g KH_2_PO_4_, 1 g NH_4_Cl, 0.5 g NaCl, 2 mL MgSO_4_ 1M, 0.1 mL CaCl_2_ 1M, 3 mL trace element solution (2.3 g/L FeCl_3_-6H_2_O, 0.039 g/L CuSO_4_-5H_2_O, 0.049 g/L ZnSO_4_-7^.^H_2_O, 0.32 g/L MnCl_2_-7^.^H_2_O, 0.129 g/L CoCl_2_-6H_2_O, 0.037 g/L (NH_3_)_6_Mo_7_O_24_-4^.^H_2_O and 0.25 g/L H_3_BO_3_). Media components were purchased from Panreac Química SLU (Barcelona, Spain). The medium was supplemented with 2 g/L of NaHCO_3_ (sodium hydrogen carbonate) and 1 mL of de thiamine 1 mg/mL. M9 medium contained 10.5 ± 0.5 g/L of glycerol for the screening of overexpression enzymes and 10.5 ± 0.5 or 12.5 ± 0.5 g/L of glycerol for optimization of the medium using different bicarbonate concentrations (0, 2, 4 and 6 g/L). Minimal mediums with 13 g/L of glucose and 2 and 4 g/L of bicarbonate were also assayed. *E. coli* strains were initially streaked from −80°C glycerol stocks on Luria-Bertani (LB) agar plates. Pre-inoculum was prepared by transferring a colony to 5 mL of LB broth and incubated overnight by adding kanamycin (for overexpression in pBAD-18-kan plasmid), or ampicillin (for overexpression in pBAD/His A plasmid) to agar plates and pre-inoculla to obtain final concentrations of 50 and 100 µg/mL, respectively. Antibiotics were purchased from Sigma-Aldrich (Merck KGaA, Darmstadt, Germany). For screening assays of anaplerotic and cataplerotic enzyme overexpressions, pre-inoculum was transferred into 15 mL of M9 medium with 10.5 g/L of glycerol and incubated overnight with the corresponding antibiotic; in the case of the evaluation of culture medium components, pre-inoculum was incubated overnight and then transferred into 50 mL of culture medium with 12.5 g/L of glycerol without antibiotics. This inoculum was used to obtain an aerobic culture in 250 mL Erlenmeyer flasks containing 50 mL of M9 medium with an initial concentration of 0.023 g CDW/L for the screening of the overeexpression’s enzymes and 0.03 g CDW/L for culture media components evaluation. Fermentation was performed at 37 °C in a rotary shaker (Comecta, S.A., Barcelona, Spain) at 200 rpm up to 72 h; samples were taken at 0, 10, 24, 31, 48, 55 and 72 h. The induction of gene expressions in pBAD vectors were performed when cultures arose at OD= 0.3, which was at around 8–10 h from inoculation.

Complementation of *ppc* deletion in the M6 mutant was analyzed by cultivation 200 µL of culture medium of M4-Δ*iclR*, M6, M6/pBA-*ppc* and M6/pBAD-*pck* strains in a 500 µL of wells microplate at 30 °C for 48 h and measuring the O.D. at 570 nm of wavelength using a Multiskan^TM^ FC microplate photometer (Thermo Scientific, CA, USA).

### 4.4. Evaluation of the Influence of Culture Medium Components on Malate Production

Different culture media conditions were evaluated; firstly, a Plackett–Burman design (PBD) [35] was used to identify the independent variables that significantly influenced the malic acid molar yield (mol malic acid per mol glycerol consumed) as a response variable in the M4-Δ*iclR*/pBA-*pck* mutant strain. A total of 11 factors were included in the screening design, which were evaluated in three levels as shown in Table 1. The coded variables were defined according to:

$$x_{i}=\;\frac{2\left(C_{i}-\bar{C}\right)}{\left(C_{max}-C_{min}\right)}$$

where *C_i_* represents each component studied, varying in the interval *C_min_* − *C_max_*. Each variable is transformed into a *t*-statistic by dividing it by its standard error. Standardization values are graphed in decreasing order of the absolute magnitude with a 95% confidence interval and represented in a Pareto diagram. The experimental design and data analysis was carried out using Statgraphics Centurion [36].

L-arabinose assays were performed using 0.01, 0.025, 0.05, 0.075 and 0.1% concentrations added at O.D. = 0.6 (Table S2). The study of bicarbonate feeding was carried out using different initial concentrations, from 0 to 6 g/L of NaHCO_3_. Additionally, four different fed-batches were analyzed using an initial concentration of 2 g/L of NaHCO_3_ with an addition of bicarbonate at several time points. The effect of glycerol concentration on malic acid production was evaluated using different initial concentrations in the culture medium and a kinetic study with different glycerol concentrations (3.5, 6.75, 13.5, 20, 26.5, 31.5 and 37.5 g/L) was performed.

### 4.5. Analytical Techniques

Succinic acid, acetic acid, malic acid and glycerol were measured from a filtered culture supernatant by High Performance Liquid Chromatography (HPLC), as described in [37]. The optical density (O.D.) was measured with a Spectroquant® Pharo UV/Vis spectrophotometer (Merck KGaA, Darmstadt, Germany) HITACHI Instruments Inc. Tokyo) at 570 nm of the wavelength and used to estimate the CDW (1 OD_570_ = 0.33 g of cell dry weight [CDW]/L). The volumetric production, yield and specific production and productivity were calculated as described: volumetric production: C4 (mM); molar yield: C4 production/glycerol consumed (mol/mol); specific production: C4 (mmol) × g CDW^−1^; and specific productivity: C4 (mmol) × g CDW^−1^ h^−1^. Statistical analysis was carried using Statgraphics Centurion [36] and SigmaPlot [38]. Determination of total carbon was performed using the M4Δ*iclR*/pBA-*pck* strain grown in the culture medium with 12.5 g/L of glycerol and 4 g/L of NaHCO_3_, as described above. To this end, 47 mL of the cell culture at 48 h was centrifuged at 5000 × g for 10 min. The supernatant was transferred into a glass tube, and the biomass was washed with water and transferred into another glass tube. Both tubes were dried in a muffle at 105 °C for 72 h until the remaining water evaporated. For each sample, 5–10 mg were used for C elemental analysis using the X-ray fluorescent (XRF) S4 Piooner (Bruker AXS, GmbH Karlsruhe, Germany) facilities at the Central Service of Science and Technology (SC-ICYT-University of Cadiz).

## 5. Conclusions

In conclusion, we have demonstrated that *E. coli* can biotransform glycerol to malate under aerobic conditions through metabolic engineering of the TCA cycle and overexpression of the autologous Pck. The maximum malate production obtained was 57.5 % of the theoretical maximum molar yield, achieved with the supplementation of 4 g/L of sodium hydrogen bicarbonate and using 12.5 g/L of glycerol. The production allowed us to obtain 5.2 g/L of malate and 2 g/L of succinate as by-products. These results highlight the importance not only of the rewiring of *E. coli* metabolism but also of pH and bicarbonate addition for enhancing malate production. This work establishes a basis for further optimization of malate production from glycerol, which could be achieved by avoiding acetate leakage by diverting the carbon flux from succinate to malate and by improving malate export.

## Figures and Tables

**Table 1:** Medium factors and the levels used in Plackett-Burman design (PBD). The factor levels are described as central (0), higher (+1) and lower (–1**).** The studied combinations of the medium factors are included in Supplementary Material Table S1.

FACTORS	+1	0	−1
Na_2_HPO4 (g/L)	12	10	8
KH2PO4 (g/L)	10	8	6
NaHCO3 (g/L)	4	2	0
NaCl (g/L)	1	0.5	0
NH4Cl (g/L)	3	1.75	0.5
Glycerol (g/L)	19.5	13.5	6.5
MgSO4 1M (mL/L)	4	2.25	0.5
CaCl_2_ 1M (mL/L)	0.15	0.1	0.05
Trace elements (mL/L)	0.15	0.075	0
Thiamine (mL/L)	1.5	0.75	0
L-arabinose (% m/V)	0.02	0.0101	0.0002

## Data Availability

The data presented in this study are available in the article or Supplementary Materials.

## Supplementary Materials

The following are available online at https://www.mdpi.com/1422-0067/22/5/2266/s1.
