# Supplementary material for: Identification of Enzymatic Bottlenecks for the Aerobic Production of Malate from Glycerol by the Systematic Gene Overexpression of Anaplerotic Enzymes in Escherichia coli
Source: Int J Mol Sci. 2021 Feb 25;22(5):2266. doi: 10.3390/ijms22052266 (PMC7956688; doi:10.3390/ijms22052266)
Supplement: Supplementary file 1 [file ijms-22-02266-s001.pdf]

Supplementary materials

Fig. S1. Cumulative bar charts of C4 metabolites and acetate productions yield (g/g) of overexpression and co-expression on M4 and M4-*ΔiclR* strains at 48 h. Malate, succinate, and acetate. Standard deviation of the variables plotted in this figure is shown in Supplementary Material Table S3.

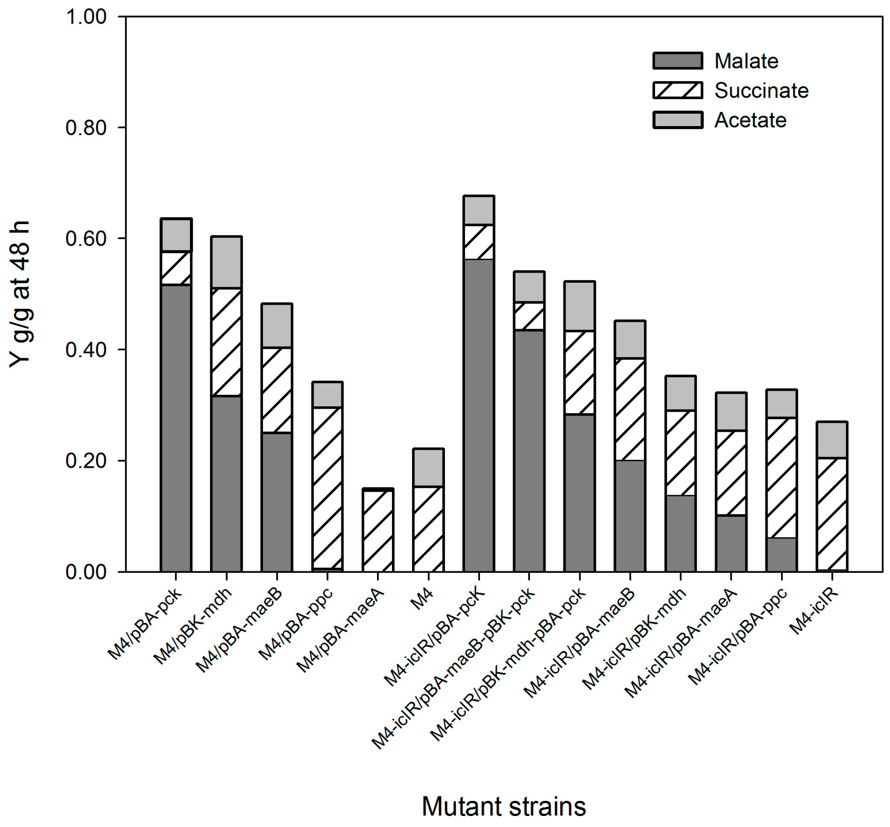

Fig. S2. Analysis of the M4-Δ*iclR*/pB-*pck* strain growing on M9 with increasing glycerol concentrations. (A) Curves of growth in g CDW/ L; (B) residual glycerol in the culture medium; and (C) concentration of malate g/L. The samples were taken at 0, 10, 24, 31, and 48 h.

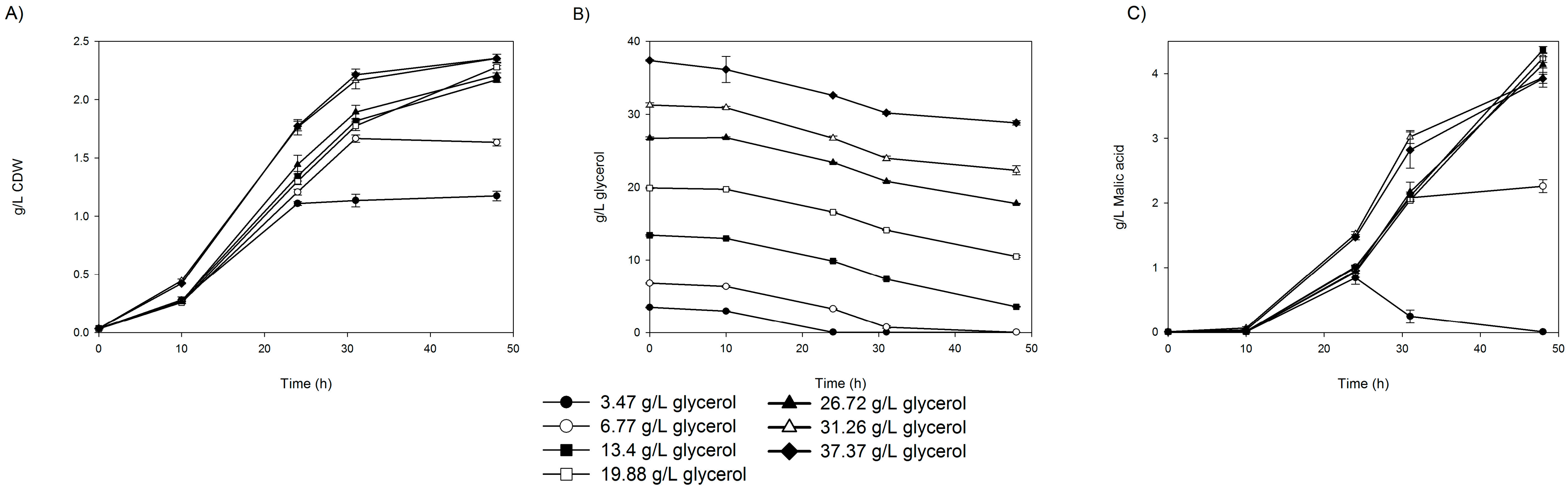

Fig. S3. Assay of M4-*ΔiclR/*pBA-*pck* strain grown in M9 medium containing 12.5 g/L glycerol. A) 0 g/L of bicarbonate or B) 6 g/L of bicarbonate. The variables measured were in (g/L): malic acid, succinic acid, acetic acid, glycerol, in (g CDW/L) biomass and pH. The samples were taken at 0, 10, 24, 31, 48, 51, 72 and 96 hours. Standard deviation of the parameters evaluated are indicated in Table S3.

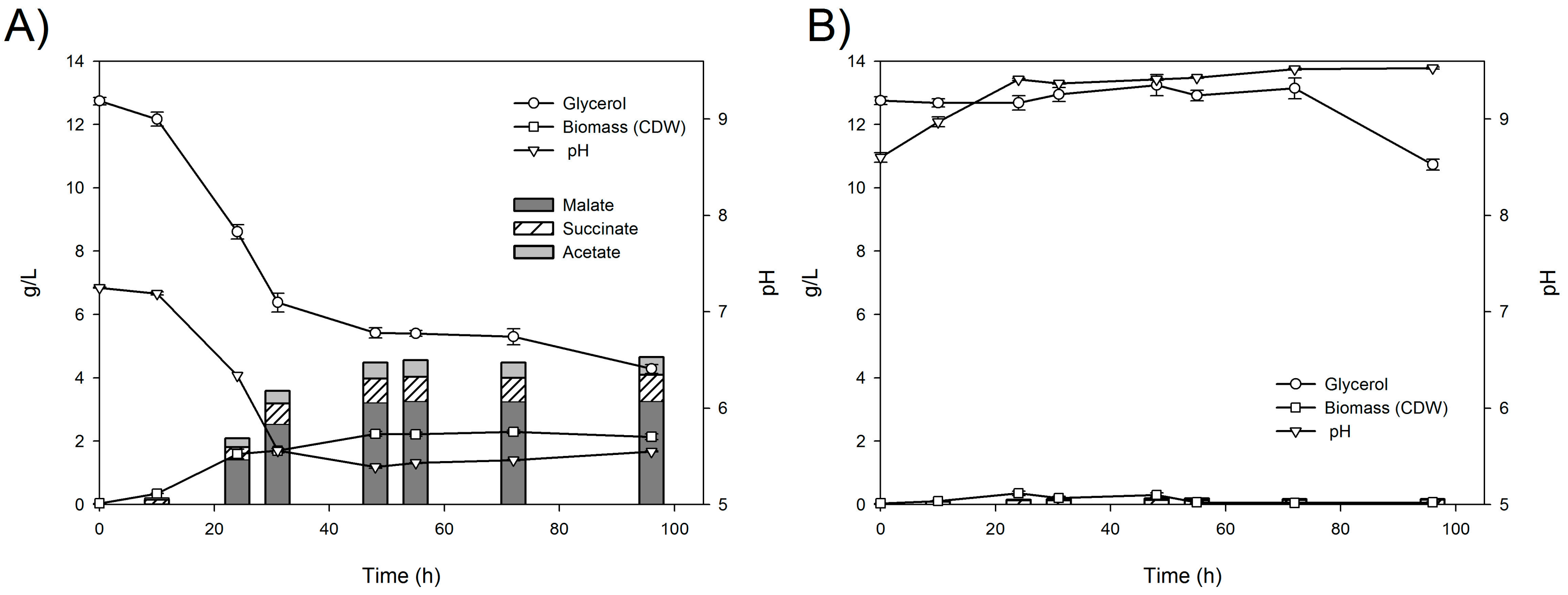

Fig. S4. Assay of M4-*ΔiclR/*pBA-*pck* strain grown in M9 medium containing 12.5 g/L glycerol and different bicarbonate feedings. A) Feeding 1: 1 g/L of bicarbonate at 10, 24, 31 and 48 h; B) Feeding 2: 2 g/L of bicarbonate at 24 and 31 h. C) Feeding 3: 2 g/L of bicarbonate at 24 and 48 h. D) Feeding 4: 2 g/L only at 24 h. The variables measured were in (g/L): malic acid, succinic acid, acetic acid, glycerol, in (g CDW/L) biomass and pH. Standard deviation are shown in Supplementary Material Table S7.

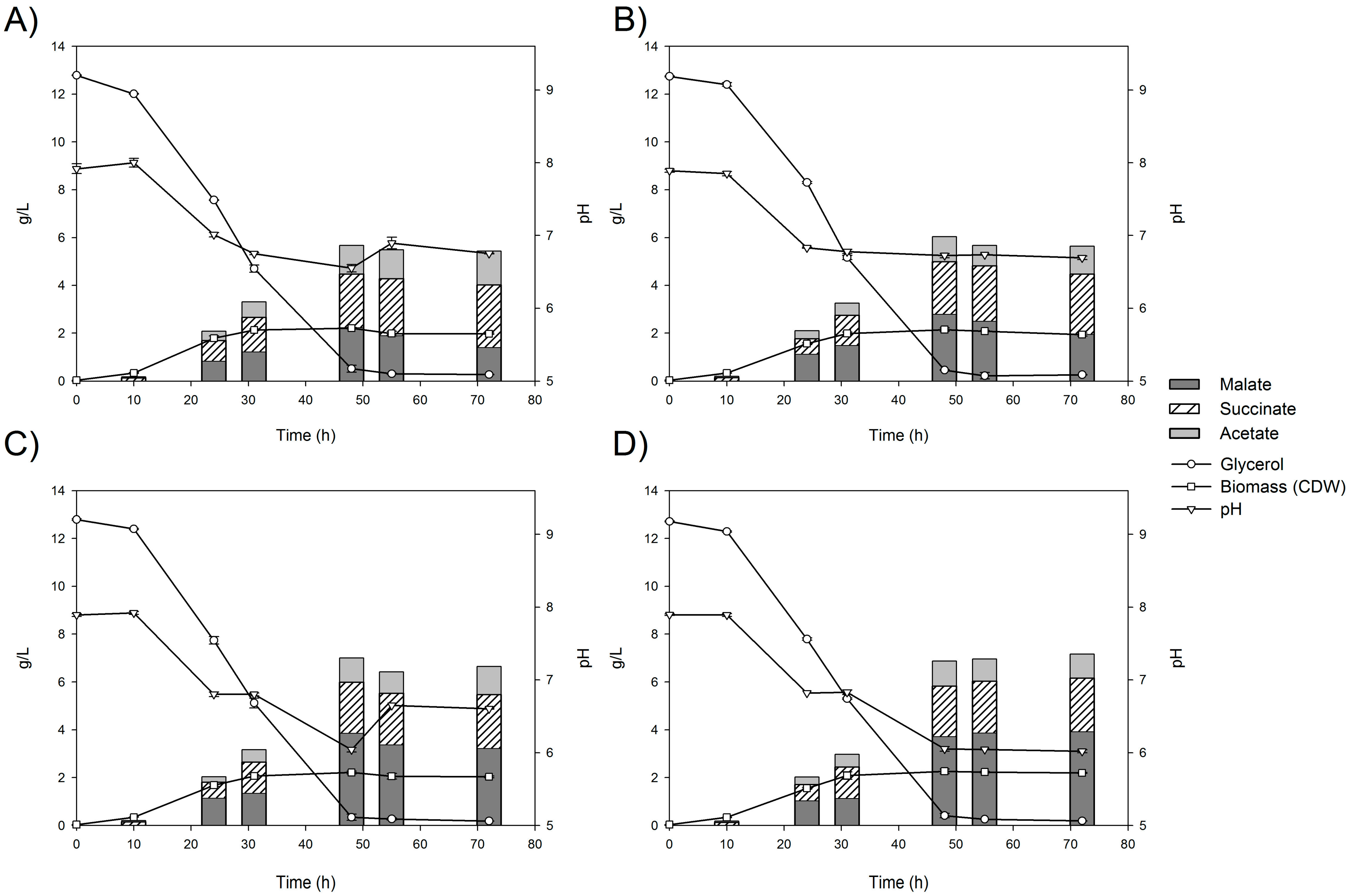

Fig. S5. Assay of M4-*ΔiclR/*pBA-*pck* strain strain grown in M9 medium containing 12.5 g/L glycerol and 2 g/L bicarbonate with pH control. In this assay pH was adjusted to 5.85 with NaOH at hours 24 and 31 h. The variables measured were in (g/L): malic acid, succinic acid, acetic acid, glycerol, in (g CDW/L) biomass and pH. The samples were taken at 0, 10, 24, 31, 48, 51, 72 and 96 hours. Standard deviation of the parameters evaluated are indicated in Table S3.

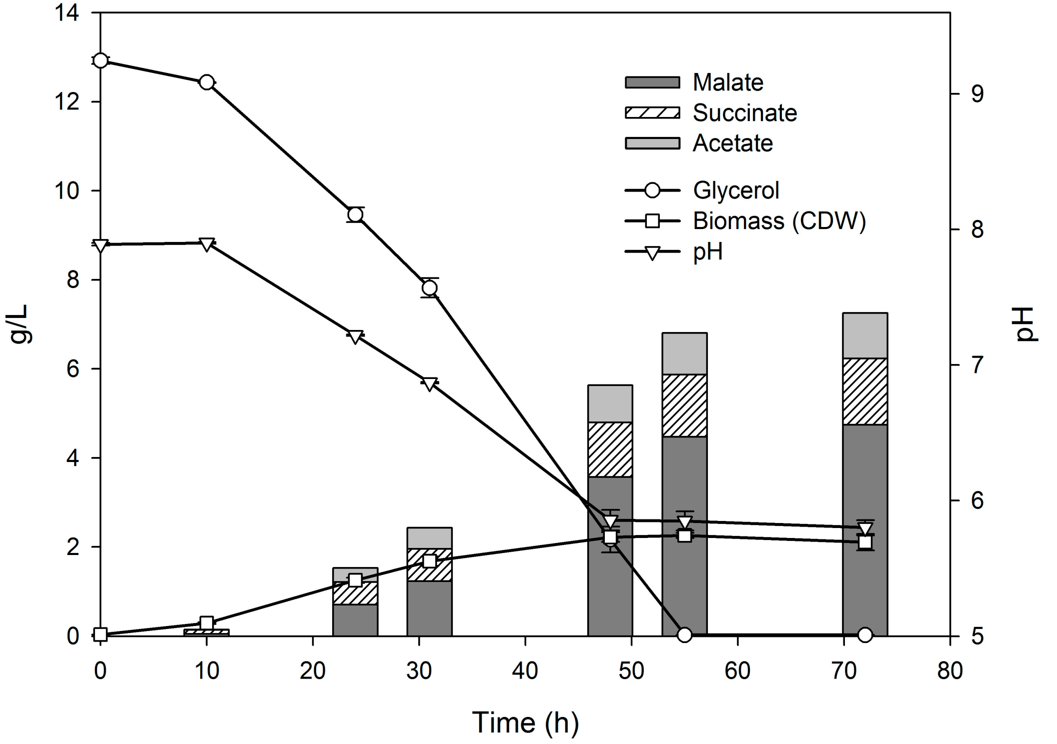

Fig. S6. Growth curves of biomass concentration g CDW/L. The strains were grown on M9 containing 12,5 g/L glycerol and 4 g/L bicarbonate. The strains in this assays are: M6 (M4-Δ*iclR*Δ*ppc*), M4-*ΔiclR*, M6/pBA-*pck* and M6/pBA-*ppc* (Δ*ppc* complementation). Each point represents the average and error bars the standard deviations of three biological replicates.

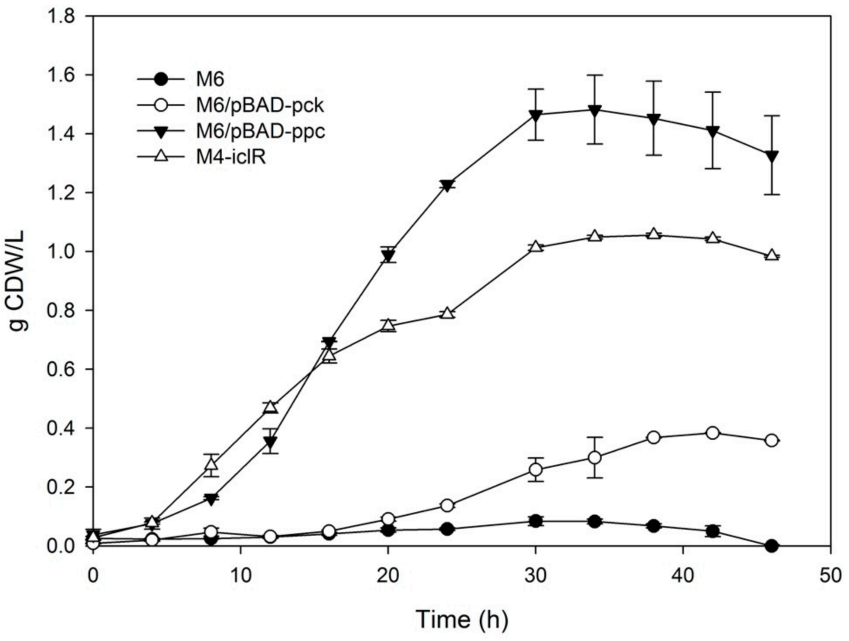

Fig. S7. Proposed rewiring of the metabolic flux in the M4 strain after further modifications. A) Effects of the activation of the glyoxylate shunt (Δ*iclR*). B) Effect of *maeB* overexpression. C) Effect of *maeA* overexpression

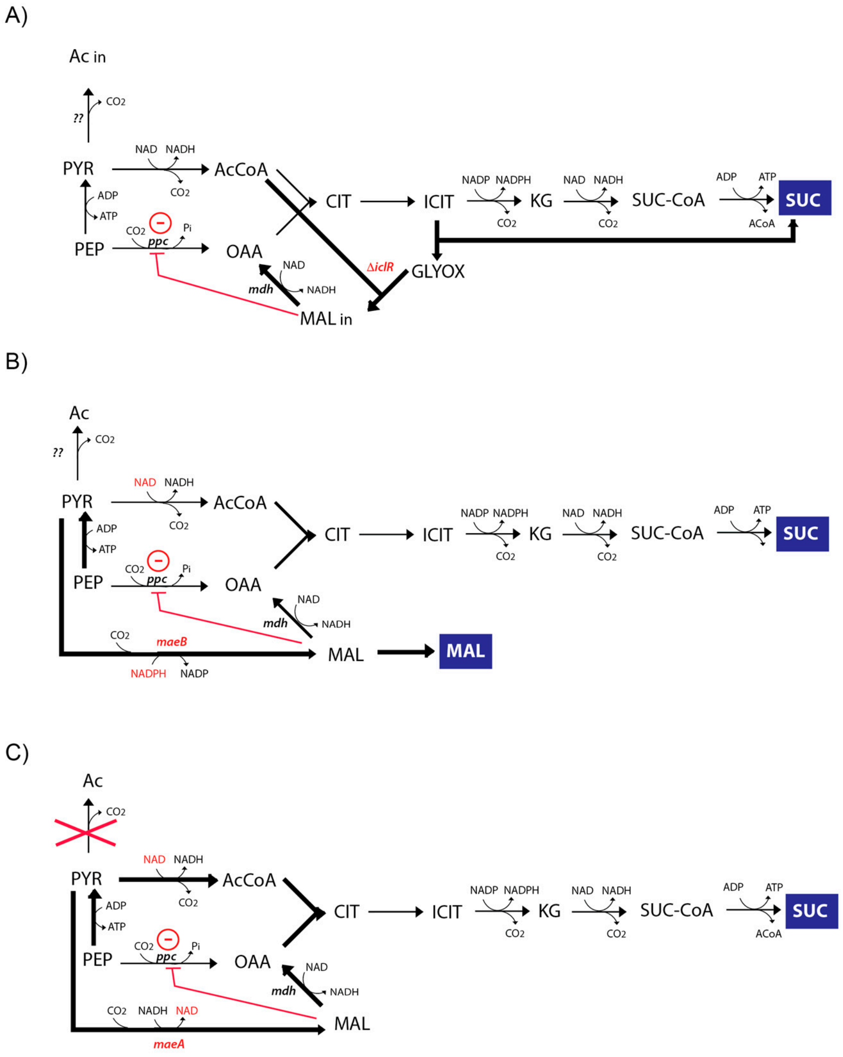

Table S1. Table of combinations of the medium factors and calculation of response variable for each run

	FACTORS											
	**A**	**B**	**C**	**D**	**E**	**F**	**G**	**H**	**I**	**J**	**K**	**Response variable calculation**
Run 1	1	−1	1	−1	−1	−1	1	1	1	−1	1	0.2248
Run 2	1	1	−1	1	−1	−1	−1	1	1	1	−1	0.0000
Run 3	−1	1	1	−1	1	−1	−1	−1	1	1	1	0.1811
Run 4	1	−1	1	1	−1	1	−1	−1	−1	1	1	0.1972
Run 5	1	1	−1	1	1	−1	1	−1	−1	−1	1	0.3512
Run 6	1	1	1	−1	1	1	−1	1	−1	−1	−1	0.1189
Run 7	−1	1	1	1	−1	1	1	−1	1	−1	−1	0.0021
Run 8	−1	−1	1	1	1	−1	1	1	−1	1	−1	0.0000
Run 9	−1	−1	−1	1	1	1	−1	1	1	−1	1	0.3143
Run 10	1	−1	−1	−1	1	1	1	−1	1	1	−1	0.1175
Run 11	−1	1	−1	−1	−1	1	1	1	−1	1	1	0.4089
Run 12	−1	−1	−1	−1	−1	−1	−1	−1	−1	−1	−1	0.0270
Run 13	0	0	0	0	0	0	0	0	0	0	0	0.2714
Run 14	0	0	0	0	0	0	0	0	0	0	0	0.2339
Run 15	0	0	0	0	0	0	0	0	0	0	0	0.3034

Table S2. Parameters calculated from assay with increasing L-arabinose concentrations.

L-arabinose (%)	Molar Yield (mol/mol)			Productivity (mol x h^{-1})			Specific production (mmol x g CDW^{-1})			Specific productivity (mmol x g CDW^{-1} x h^{-1})		
	**24 h**	**48 h**	**72 h**	**24 h**	**48 h**	**72 h**	**24 h**	**48 h**	**72 h**	**24 h**	**48 h**	**72 h**
**0.01**	0.26 ± 0.00	0.40 ± 0.01	0.40 ± 0.01	0.43 ± 0.01	0.70 ± 0.02	0.49 ± 0.01	6.21 ± 0.24	14.53 ± 0.25	12.72 ± 0.15	0.26 ± 0.01	0.30 ± 0.01	0.18 ± 0.00
**0.025**	0.32 ± 0.01	0.42 ± 0.01	0.43 ± 0.01	0.38 ± 0.01	0.69 ± 0.02	0.48 ± 0.01	7.82 ± 0.37	17.49 ± 0.64	13.40 ± 1.11	0.33 ± 0.02	0.36 ± 0.01	0.19 ± 0.02
**0.05**	0.33 ± 0.01	0.43 ± 0.02	0.44 ± 0.01	0.33 ± 0.00	0.69 ± 0.02	0.49 ± 0.00	7.20 ± 0.12	18.97 ± 0.55	16.30 ± 0.47	0.30 ± 0.00	0.40 ± 0.01	0.23 ± 0.01
**0.075**	0.31 ± 0.02	0.34 ± 0.03	0.37 ± 0.01	0.29 ± 0.02	0.53 ± 0.06	0.41 ± 0.03	6.83 ± 0.10	15.89 ± 1.76	15.02 ± 1.13	0.28 ± 0.00	0.33 ± 0.04	0.21 ± 0.02
**0.1**	0.32 ± 0.02	0.36 ± 0.04	0.36 ± 0.01	0.28 ± 0.01	0.48 ± 0.04	0.40 ± 0.02	6.84 ± 0.19	14.89 ± 0.71	15.39 ± 0.40	0.28 ± 0.01	0.31 ± 0.01	0.21 ± 0.01

Table S3. Standard deviation of assay with increasing bicarbonate concentration; NaOH and glucose with 4 g/L bicarbonate.

Hour	0 g/L			2 g/L			4 g/L			6 g/L			NaOH			Glucose		
	**Suc**	**Mal**	**Ace**	**Suc**	**Mal**	**Ace**	**Suc**	**Mal**	**Ace**	**Suc**	**Mal**	**Ace**	**Suc**	**Mal**	**Ace**	**Suc**	**Mal**	**Ace**
**0**	0.00	0.00	0.00	0.00	0.00	0.00	0.00	0.00	0.00	0.00	0.00	0.00	0.00	0.00	0.00	0.00	0.00	0.00
**10**	0.00	0.01	0.01	0.00	0.00	0.00	0.00	0.00	0.01	0.01	0.00	0.00	0.01	0.00	0.00	0.03	0.02	0.01
**24**	0.02	0.03	0.00	0.02	0.05	0.01	0.02	0.02	0.02	0.01	0.00	0.00	0.03	0.11	0.11	0.08	0.21	0.02
**31**	0.05	0.06	0.00	0.01	0.04	0.00	0.03	0.04	0.00	0.01	0.00	0.00	0.06	0.22	0.22	0.08	0.24	0.03
**48**	0.03	0.04	0.01	0.65	0.04	0.01	0.02	0.05	0.00	0.01	0.00	0.00	0.10	0.27	0.26	0.09	0.26	0.07
**55**	0.03	0.04	0.00	0.07	0.11	0.03	0.02	0.07	0.04	0.02	0.00	0.00	0.11	0.41	0.41	0.13	0.00	0.08
**72**	0.05	0.16	0.10	0.06	0.15	0.02	0.01	0.10	0.00	0.01	0.00	0.00	0.15	0.38	0.37	0.03	0.01	0.05
**96**	0.03	0.09	0.01	0.06	0.13	0.02	0.02	0.15	0.01	0.01	0.00	0.00	0.14	0.20	0.30	0.03	0.01	0.10

Table S4. Elemental C analysis measured at 48 h since inoculation of M4-Δ*iclR*/pBA-*pck* and g/L C estimated through quantification of analytes by HPLC.

		**0 h**	**48 h**		
**Culture media conditions**	**Separation culture media**	**g/L Carbon**	**% C [c]**	**g/L C calculated by elemental analysis [c]**	**g/L C estimated by HPLC analysis [d]**
12.5 g/L glycerol and 4 g/L $NaHCO_3$	Biomass	0.030±0.000[a]	45.06±0.20	0.66±0.00	---
	Supernatant[b]	5.610±0.003[b]	14.10±1.26	2.96±0.28	2.99±0.10

[a] Measurement since elemental analysis of C (37.6%) and biomass weight of preculture.

[b] Measurement since calculation of g/L and content of mol C of glycerol, HCO_3 and thiamine added in the culture medium

[c] Average and standard deviation calculated with two analytical replicates of three biological replicates.

[d] The analytes measured by HPLC in the supernatant are: remained glycerol, malate, succinate and acetate from three biological replicates.

Table S5. List of primers sequences for knock out, check de gene deletions and for cloning. In italics are denoted the enzyme restriction for directed cloning in 5' (PF) and 3' (PR), in lower case the enzyme restriction sequence within the primer and in bold the initial codon of Methionine (ATG) and STOP codon (TTA).

Primers	Sequence 5'→3'	Source or reference
poxB H1P4	GATGAACTAAACTTGTTACCGTTATCACATTCAGGAGATGGAGAACCATGATTCCGGGGATCCGTCGACC	Coli Genetic Stock Center (CGSC). University of Yale
poxB H2P1	CCTTATTATGACGGGAAATGCCACCCTTTTTACCTTAGCCAGTTTGTTTTGTGTAGGCTGGAGCTGCTTC	CGSC
ack-pta H1P4	TGGCTCCCTGACGTTTTTTTAGCCACGTATCAATTATAGGTACTTCCATGATTCCGGGGATCCGTCGACC	CGSC
ack-pta H2P1	GCAGCGCAAAGCTGCGGATGATGACGAGATTACTGCTGCTGTGCAGACTGGTGTAGGCTGGAGCTGCTTC	CGSC
iclR H1P4	CAATAAAAATGAAAATGATTTCCACGATACAGAAAAAAGAGACTGTCATGATTCCGGGGATCCGTCGACC	CGSC
iclR H2P1	AGAATATTGCCTCTGCCCGCCAGAAAAAGTCAGCGCATTCCACCGTACGCGTGTAGGCTGGAGCTGCTTC	CGSC
ppc-H1P4	GAAGGATACAGGGCTATCAAACGATAAGATGGGGTGTCTGGGGTAATATGATTCCGGGGATCCGTCGACC	CGSC
ppc-H2P1	AAAGCACGAGGGTTTGCAGAAGAGGAAGATTAGCCGGTATTACGCATACCTGTAGGCTGGAGCTGCTTCG	CGSC
sdhA-F	GAACAGCCTATACTGCCGCC	This study
sdhA-R	TTTATGCTTACTTCGCCGTGG	This study
pox-F	GGCTGCTGTAAGACAAAAGTGG	This study
pox-R	TCAAACAGATAGTTATGCGCGG	This study
ack-pta-F	ATTATCCGGCGTTGACATGC	This study
ack-pta-R	TGGATCTACGACTTTGCGTG	This study
iclR-F	TTTGCTGCTCACACTTGCTC	This study
iclR-R	GGTGTTCATTTGTCTGGGCTG	This study
ppc-F	CAGTGACTCAAACGATGCCC	This study
ppc-R	CCTGGACTTCTGTGGAATGC	This study
Kt	CGGCCACAGTCGATGAATCC	[31]
pBA-*NheI*-ppc-PF	GGgctagc**ATG**AACGAACAATATTCCGCATTGC	[34]
pBA-*EcoRI*-ppc-PR	GGgaattc**TTA**GCCGGTATTACGCATACCTG	[34]
pBA-*NheI*-pck-PF	GGgctagc**ATG**CGCGTTAACAATGGTTTGAC	[34]
pBA(K)-*EcoRI*-pck-PR	GGgaattc**TTA**CAGTTTCGGACCAGCCGC	[34]
pBK-*NheI*-pck-PF	GGgctagcAGGAGGAATTAACC**ATG**CGCGTTAACAATGGTTTGAC	This study
pBA-*NcoI*-maeB-PF	GGGccatgg**ATG**ACCAGTTAAAACAAAGTG	This study
pBA-*HindIII*-maeB-PR	GGGaagctt**TTA**CAGCGGTTGGGTTTGC	This study
pBA-*NheI*-maeA-PF	CCCgctagc**ATG**GAACCAAAAACAAAAAAACAGCG	This study
pBA-*HindIII*-maeA-PR	CCCaagctt**TTA**GATGGAGGTACGGCGGTAG	This study
pBK-*EcoRI*-mdh-PF	GGGgaattcAGGAGGAATTAACC**ATG**AAAGTCGCAGTCCTCGG	This study
pBK-*XbaI*-mdh-P	GGGtctaga**TTA**CTTATTAACGAACTCTTCGCC	This study

Table S6. List of plasmid names, features and sources.

Plasmids	Features	Source
pKD13	P4-FRT-kan-FRT-P1 with *bla* (β-lactamase gene) that conferring ampicillin resistance	Coli Genetic Stock Center (CGSC). University of Yale
pCP20	cat, yeast *Flp* recombinase with *bla* gene	CGSC
pKD46	*bla gene,* k-Red recombinase under araBAD promoter, temperature-conditional replicon	CGSC
pBAD/His-A	P_{BAD}-SD-ATG-MCS--myc--6xHis—Term under arabinose promoter (P_{BAD}) with *bla* gene	Invitrogen Ref (V430-01)
pBAD18-Kan	P_{BAD}--ATG-Term with kanamycin resistance gene	Jonathan Beckwith (Harvard Medical School) [32]
pBA-pck	pBAD/His A vector cloning with phosphoenol pyruvate carboxykinase ORF (*pck)*	[34]
pBK-pck	pBAD-18-kan vector cloning with phosphoenol pyruvate carboxykinase ORF (*pck)*	This study
pBK-mdh	pBAD-18-kan vector cloning with malate dehydrogenase ORF (*mdh)*	This study
pBA-maeA	pBAD/His A vector cloning with NAD^+ dependent malic enzyme ORF (*maeA*)	This study
pBA-maeB	pBAD/His A vector cloning with $NADP^+$ dependent malic enzyme ORF (*maeB*)	This study
pBA-ppc	pBAD/His A vector cloning with phosphoenol pyruvate carboxylase ORF (*ppc)*	[34]

Table S7. List of engineered strains constructed in this work and the parental strain.

Strains	Genotype	Source or reference
BW25113	K12 *F*⁻, Δ(*araD-araB*)567, Δ*lacZ*4787(::*rrnB*-3), λ-, *rph*-1, Δ(*rhaD-rhaB*)568, *hsdR514*	Keio Collection (NBRP)
M1	*E. coli K12 BW25113* Δ*sdhA*::Kan; defective in succinate dehydrogenase subunit A	Keio Collection (NBRP)
M4	M1Δ*ack-pta*::FRTΔ*pox*::kan; M1 mutant and defective of acetate kinase, phosphate acetyltransferase, and pyruvate oxidase.	This study
M4-Δ*iclR*	M4Δ*iclR*::kan M4 mutant and defective DNA-binding transcriptional repressor IclR	This study
M6	M4Δ*iclR*::FRTΔ*ppc*; defective in phosphoenol pyruvate carboxylase.	This study
M4/pBA-***pck***	Δ*sdhA*::FRTΔ*ack-pta*::FRTΔ*pox::kan* harbouring the pBAD-A-pck cloning vector	This study
M4/pBK-mdh	Δ*sdhA::*FRTΔ*ack-pta*::FRTΔ*pox::kan* harbouring the pBAD18-K-mdh cloning vector	This study
M4/pBA-maeB	Δ*sdhA::*FRTΔ*ack-pta*::FRTΔ*pox::kan* harbouring the pBAD-A-maeB cloning vector	This study
M4/pBA-ppc	Δ*sdhA::*FRTΔ*ack-pta*::FRTΔ*pox::kan* harbouring the pBAD-A-ppc cloning vector	This study
M4/pBA-maeA	Δ*sdhA::*FRTΔ*ack-pta*::FRTΔ*pox::kan* harbouring the pBAD-A-maeA cloning vector	
M4-Δ*iclR*/pBA-pck	Δ*sdhA::*FRTΔ*ack-pta*::FRTΔ*pox::*FRTΔ*iclR::*FRT harbouring the pBAD-A-pck cloning vector	This study
M4-Δ*iclR/*pBA-maeB+pBK-pck	Δ*sdhA::*FRTΔ*ack-pta*::FRTΔ*pox::*FRTΔ*iclR::*FRT harbouring the pBAD-A-maeB cloning vector and pBAD-18-Kan-pck cloning vector	This study
M4-Δ*iclR/*pBK-mdh+pBA-pck	Δ*sdhA::*FRTΔ*ack-pta*::FRTΔ*pox::*FRTΔ*iclR::*FRT harbouring the pBAD18-K-mdh cloning vector and the pBAD-A-pck cloning vector	This study
M4-Δ*iclR/*pBA-maeB	Δ*sdhA::*FRTΔ*ack-pta*::FRTΔ*pox::*FRTΔ*iclR::*FRT harbouring the pBAD-A-maeB cloning vector	This study
M4-Δ*iclR/*pBK-mdh	Δ*sdhA::*FRTΔ*ack-pta*::FRTΔ*pox::*FRTΔ*iclR::*FRT harbouring the pBAD18-K-mdh cloning vector	This study
M4-Δ*iclR*/pBA-maeA	Δ*sdhA::*FRTΔ*ack-pta*::FRTΔ*pox::*FRTΔ*iclR::*FRT harbouring the pBAD-A-maeA cloning vector	This study
M4-Δ*iclR*/pBA-ppc	Δ*sdhA::*FRTΔ*ack-pta*::FRTΔ*pox::*FRTΔ*iclR::*FRT harbouring the pBAD-A-ppc cloning vector	This study
